# Transvection-Based Gene Regulation in *Drosophila* Is a Complex and Plastic Trait

**DOI:** 10.1534/g3.114.012484

**Published:** 2014-09-11

**Authors:** Xinyang Bing, Teresa Z. Rzezniczak, Jack R. Bateman, Thomas J. S. Merritt

**Affiliations:** * Department of Chemistry and Biochemistry, Laurentian University, Sudbury, ON, P3E 2C6, Canada; † Biology Department, Bowdoin College, Brunswick, Maine 04011

**Keywords:** transvection, *trans*-interactions, *Malic enzyme* (*Men*), *Abdominal-B* (*Abd-B*), phenotypic plasticity, genotype by environment interactions, *Drosophila melanogaster*

## Abstract

Transvection, a chromosome pairing-dependent form of *trans*-based gene regulation, is potentially widespread in the *Drosophila melanogaster* genome and varies across cell types and within tissues in *D. melanogaster*, characteristics of a complex trait. Here, we demonstrate that the *trans*-interactions at the *Malic enzyme* (*Men*) locus are, in fact, transvection as classically defined and are plastic with respect to both genetic background and environment. Using chromosomal inversions, we show that *trans*-interactions at the *Men* locus are eliminated by changes in chromosomal architecture that presumably disrupt somatic pairing. We further show that the magnitude of transvection at the *Men* locus is modified by both genetic background and environment (temperature), demonstrating that transvection is a plastic phenotype. Our results suggest that transvection effects in *D. melanogaster* are shaped by a dynamic interplay between environment and genetic background. Interestingly, we find that *cis*-based regulation of the *Men* gene is more robust to genetic background and environment than *trans*-based. Finally, we begin to uncover the nonlocal factors that may contribute to variation in transvection overall, implicating *Abd-B* in the regulation of *Men* in *cis* and in *trans* in an allele-specific and tissue-specific manner, driven by differences in expression of the two genes across genetic backgrounds and environmental conditions.

Genome function is regulated through linear DNA sequence and three-dimensional chromatin conformation (reviewed by Cavalli and Misteli 2013). Gene expression, an important part of genome function, is largely regulated through local interactions between elements on the same chromosome (*cis*-interactions). However, interactions between elements on separate chromosomes, *trans*-interactions, can substantially modify gene expression (reviewed by Bartkuhn and Renkawitz 2008; Cavalli and Misteli 2013; Williams *et al.* 2010). Some *trans*-interactions appear to be dependent on physical pairing of homologous chromosomes or regions of chromosomes. Interest in pairing-dependent gene regulation, or misregulation, has been fueled by the implication of pairing-dependent *trans*-interactions in regulation of gene expression both during normal cell development (Bacher *et al.* 2006; Xu *et al.* 2006) and in various disease states (Koeman *et al.* 2008; Liu *et al.* 2008; Thatcher *et al.* 2005).

*Drosophila melanogaster* is an excellent model system for studying the role of pairing-dependent *trans*-interactions in gene regulation. Homologous chromosomes in *Drosophila* are extensively paired in the somatic nucleus of all cell types (reviewed by McKee 2004), and pairing-dependent *trans*-interactions, or at least the potential for these interactions, are widespread in the *Drosophila* genome (Bateman *et al.* 2012; Chen *et al.* 2002; Mellert and Truman 2012). Referred to as transvection (Lewis 1954), pairing-dependent *trans*-interactions can lead to either activation or inhibition of gene expression (reviewed by Duncan 2002; Kennison and Southworth 2002). Many cases of gene activation by transvection involve intragenic complementation between two loss-of-function or hypomorphic alleles in a pairing-dependent manner (enhancer action in *trans*; *e.g.*, Leiserson *et al.* 1994; Lewis 1954), although the phenomenon can also involve looping of insulators and enhancers (Morris *et al.* 1998, 1999). Despite extensive study of transvection in *Drosophila*, a comprehensive model of the molecular mechanisms of these pairing-dependent *trans*-interactions is still being developed.

The *D. melanogaster Malic enzyme* (*Men*) gene is developing into a promising system for determination of the molecular mechanisms underlying *trans*-interactions. Flies heterozygous for small deletion knockout alleles of the *Malic enzyme* gene (*Men*) have greater than expected levels of malic enzyme (MEN) protein activity that are not simply physiological up-regulation (Merritt *et al.* 2005; 2009; Lum and Merritt 2011; Figure 1). Lum and Merritt (2011) used a suite of knockout alleles (*MenExi^−^*) with small deletions around the *Men* transcription start site (Figure 1, B and C) and a set of *Men^+^* third chromosomes extracted from wild populations to demonstrate that the high levels of MEN activity were driven by *trans*-interaction−dependent up-regulation, and that the amount of increased MEN activity varied with the size and location of the excisions and the genetic background of the fly. The authors suggested that the up-regulation resulted from transvection (Figure 1, D and E). Although the *Men* regulatory region has not been well characterized, Lum and Merritt (2011) identified a suite of potential regulatory sites in the region computationally and suggested that the experimental differences *in trans*-effects of alleles with even small differences in their excision may be a function of multiple interacting regulatory sites. Interestingly, the differences in *trans*-effects caused by different deletion alleles and third chromosomes backgrounds often were subtle, and the significant variations were only detectable because of the sensitivity of the MEN activity assay; activity differences as small as 5% can be reliably distinguished (Merritt *et al.* 2005, 2009; Lum and Merritt 2011; Rzezniczak and Merritt 2012).

**Figure 1:**
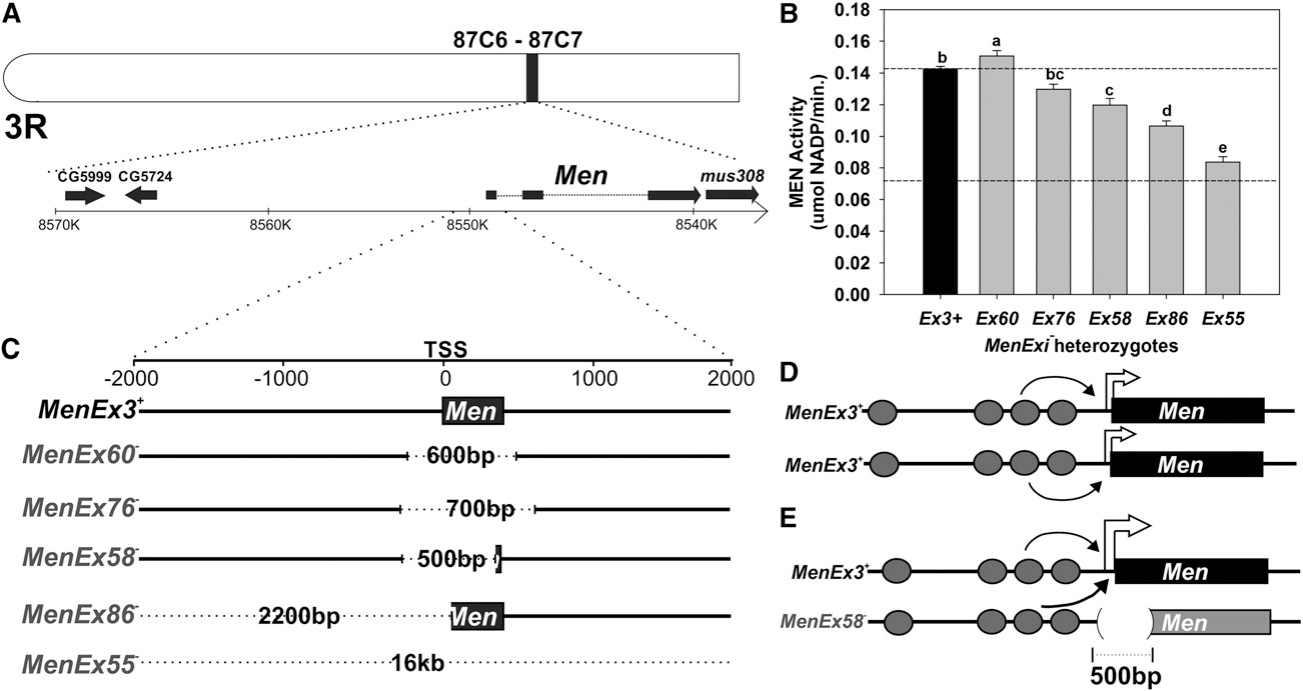
Model of *trans*-interactions at the Malic enzyme (Men). (A) The *D. melanogaster* Men locus is on the right arm of the third chromosome (3R) with 5′ region of ~17 kb devoid of other open reading frames. (B) Mean ± SE MEN enzyme activity of *MenExi^−^/MenExi^+^* heterozygotes. We investigated *trans*-interactions at this locus using a suite of *P*-element excision−derived knockout alleles, *MenExi^−^*, that drive greater than expected amounts of MEN activity when heterozygous with a functional copy of the *Men* gene (*MenExi^−^/ MenExi^+^*). Lowercase letters indicate statistical bins of MEN activity for the *MenExi^−^* alleles determined by Tukey’s honestly significant difference test after an analysis of covariance using wet weight as a covariate to normalize for possible differences in fly size (data and analysis from Lum and Merritt 2011). (C) Map of *MenExi^−^* allele excision sites: *MenEx3^+^* is a perfect excision (used as “normal” or wild-type), the other excision alleles have deletion sizes (represented by dotted line) that range from 500bp to 16kb around the transcription start site (TSS) of *Men* (see Table S1 for exact descriptions). (D) Model of gene regulation at *Men* with two functional alleles of the *Men* gene, interactions are predominantly *cis*-based. Ovals represent hypothetical transcription factor binding sites, and thin arrows represent interactions between transcription factors and the transcriptional machinery. (E) Model of gene regulation at *Men* with one functional and one knockout *Men* allele, interactions are now a combination of *cis* and *trans*. Potential synergistic interactions between the enhancers in *cis* and *trans* to the functional allele may lead to close to, and sometimes greater than, 100% wild-type MEN activity.

The sensitivity of the *trans*-interactions at *Men* to genetic background (Lum and Merritt 2011) suggested that transvection is sensitive to genetic variation across the genome. More generally, transvection may be a complex trait, phenotypically plastic with levels of transvection sensitive to both genetic background and environment. At least one classic study of transvection has suggested sensitivity to developmental temperature (Persson 1976), and recent work has shown that levels of transvection vary between cell types (Mellert and Truman 2012) and with local cellular environment (Bateman *et al.* 2012). In multicellular organisms, such differences in cell type and cellular environment are in a sense changes in environmental conditions (Ramani *et al.* 2012). Phenotypic plasticity likely results from many mechanisms, with a primary mechanism being global shifts in gene expression driven by changes in chromatin architecture (Gibert *et al.* 2007; Li *et al.* 2006; Tirosh *et al.* 2010; Zhou *et al.* 2012). Given the central role of nuclear architecture in *trans*-interactions including transvection, the sensitivity of the *trans*-interactions at *Men* to genetic background may reflect response to differences in gene expression elsewhere in the genome. A better understanding of how changes in the environment and genetic background can impact transvection will therefore provide insight into the plasticity of chromosomal architecture and its influence on gene regulation.

The complexity of *trans*-interactions at the *Men* locus, sensitive as they are to both local (allelic) and nonlocal (genetic background) variation, and the highly accurate MEN assay that allows detection of even subtle changes in gene expression, suggest that this system may be valuable for investigating the general mechanism of *trans*-interactions. This paper addresses three aspects of this system. First, although the observed *trans*-interactions at the *Men* locus have been proposed to be transvection, pairing dependence has not yet been demonstrated. Second, it is not known whether *trans*-interactions at the *Men* locus are sensitive to environmental variation, in addition to local genomic changes and variation in genetic background. Such sensitivity would support our proposal that transvection is a complex, plastic, phenotype. Third, the molecular mechanisms underlying the sensitivity of *trans*-interactions at *Men* to genetic background have not been determined. Lum and Merritt (2011) proposed, but did not demonstrate, that the variation in *trans*-interactions observed between alleles and genetic backgrounds may result from differential presence or absence of binding sites for particular transcription factors (TFs) on different *MenExi^−^* alleles, and differences in TF expression across backgrounds. Identifying binding sites and the TFs that contribute to the differences in *trans*-interactions observed across alleles and genetic backgrounds would help explain both the molecular mechanisms of *trans*-interactions at the *Men* locus and possibly *trans*-interactions in general.

## Materials and Methods

### Fly stocks and rearing conditions

Isothird chromosome lines were a subset of nonlethal third chromosomes extracted from isofemale lines: *CT21*, *HFL53*, *JFL12*, *MD76*, and *VT26* (see Sezgin *et al.* 2004). There are two known amino acid polymorphisms in the *Men* coding region of *D. melanogaster* and both affect MEN biochemistry (Merritt *et al.* 2005; Rzezniczak *et al.* 2012). The lines used in this experiment were selected to all have the same amino acid sequence to avoid possible confounding or complicating effects caused by combinations of these biochemically distinct alleles (Lum and Merritt 2011). *Men* excision alleles, both knockout (*MenExi^−^*) and wild-type (*MenEx3^+^*), were generated by *P*-element excision−mediated deletion and described previously (Merritt *et al.* 2005; Lum and Merritt 2011). All other lines were obtained from the Bloomington Drosophila Stock Center at Indiana University (see Supporting Information, Table S1 for a list and descriptions of all lines used). RNAi lines were acquired through the Bloomington Drosophila Stock Center and had been constructed as part of the Transgenic RNAi Project (TRiP) at the Harvard Medical School (Ni *et al.* 2011). All flies were maintained on a standard cornmeal medium at 50% humidity, a 12:12-hr light:dark cycle, and 25° (except in the temperature shift experiment, in which only temperature was varied).

Specific genotypes were created by crossing 10 five-d-old adult male flies from one line (*e.g.*, a *MenExi^−^* allele line) to 10 five-d-old adult female flies from another line (*e.g.*, a third chromosome background line). In all experiments except quantitative reverse-transcription polymerase chain reaction (qRT-PCR) and RNA interference (RNAi), each specific cross was done four times (four sets of parental flies) with two separate vials per generation (parental flies were placed in the first vial for 2 d, transferred to a second for 2 d, then discarded). For qRT-PCR and RNAi, each cross was performed three times with each again using duplicate vials. Emerging male flies were collected and held on fresh medium for 4 d posteclosion before further processing. For all enzyme activity assays, at least four samples of four flies were collected from each generation of crosses, sometimes pooled across vials, and stored at −80° until processing. For (qRT-PCR) experiments, samples were pooled across two or more vials from each separate generation of crosses, and at least three samples of 15 flies were collected, snap frozen in liquid nitrogen, and stored at −80° until needed for RNA extraction.

### Temperature shift

The *MenExi^−^* allele lines were crossed to the five isothird chromosome lines (above), emerging male flies were collected every second day, and transferred to either 21° or 29°, or kept at 25° (±1°), and held for 4 d.

### RNA inhibition of gene expression and *cis*-interactions

RNAi was accomplished using the GAL4/UAS system (Duffy 2002), by crossing a *Hsp70*-promoter driven GAL4 line to UAS lines from TRiP (specific lines used for each gene are listed in Table S1; Ni *et al.* 2011). Gal4/TRiP progeny were exposed to heat-shock (37° for 30 min) five times throughout development (1, 2, 4, 8, and 12 d after oviposition) to induce *Gal4* expression. Repeated heat-shocks have been shown to lead to greater *Hsp70*-promoter activity than single treatments (Kristensen *et al.* 2003). Flies were collected for experiments 2 hr after the last heat-shock treatment.

### Fly homogenization and enzyme activity assays

MEN activity was quantified as previously described (Lum and Merritt 2011). In brief, fly samples were weighed, homogenized, and centrifuged to pellet insoluble residues. MEN activity was measured using 10 µL of whole-fly homogenate in 100 µL of assay solution. Absorbance at 320 nm was measured every nine seconds for 3 min at 25°, and activity was quantified as the slope of the line of absorbance. Each sample was assayed three times and the mean used for statistical analysis. Total soluble protein concentration in the fly homogenates was measured by the bicinchoninic acid assay using a commercially available kit and standards (Pierce, Thermo Fisher Scientific, Rockford, IL).

### RNA extraction and qRT-PCR

For each genotype, RNA was extracted from at least three groups of 15 male flies. Total RNA was isolated from flies using the RNeasy Kit (QIAGEN, Valencia, CA) according to the manufacturer’s instructions and stored at −80° until needed for reverse transcription. For each sample, one microgram of total RNA was reverse transcribed using the QuantiTect Reverse Transcription Kit (QIAGEN) according to the manufacturer’s instructions.

qRT-PCR) was performed using the Quantitect Probe PCR Master Mix (QIAGEN) on a Mastercycler ep realplex Thermal Cycler (Eppendorf, Mississauga, ON) with the following parameters: 15 min at 95°; up to 45 cycles of 15 sec at 94°, and 1 min at 60°. All analyses were performed in technical triplicate, alongside a nontemplate control. Expression of a gene in a given sample was quantified relative to the average expression of the gene across all samples in each experiment. For samples from RNAi experiments, expression was quantified relative to a combination of RNAi control lines with overexpression of a null-effect gene in the vector, and background lines without a vector (see Table S1). Gene expression was normalized to two reference genes (*Actin-79B* and *Rpl32*; see Table S2 for all genes analyzed and their respective primers/probes). All analyses were done using Biogazelle’s qbase^PLUS^ software version 2.0 (Hellemans *et al.* 2007). Primers were designed to amplify exon sequences flanking an intron, based on Flybase annotations (Table S2), using the PerlPrimer software (Marshall 2004). When annotation suggested differential splicing, primers and probes were designed to match exons present in all putative splice variants.

### Data analysis

In all cases, crosses were replicated in multiple vials and multiple samples were taken from each vial. Multivariate analysis of variance tests were conducted to ascertain possible significant differences in MEN activity across genotypes or environmental conditions (following Merritt *et al.* 2005; Rzezniczak and Merritt 2012). Sample wet weight and the protein concentration of each homogenate were used as covariates in statistical analyses (protein concentration) of MEN activity to account for differences in fly size (wet weight) and degree of homogenization. Analysis of covariance and Tukey’s honestly significant difference multiple-comparison tests were performed using JMP version 7 software (SAS Institute Inc., Cary, NC, 1989−2007).

Specifically, we used a standard least squares fitting model to translate nominal groups (genotype, *Men* excision allele, temperature) into linear models. As an example, in the environmental effects experiment, y(i) = observed MEN activity of the i^th^ trial, β0 = average over all levels (intercept/grand mean), β_i_ = predictor variable i. Since temperature has three groups, 25°, 21°, and 29°, there were two β variables, β_1_ and β_2_; these β variables are the treatment effect of the three temperatures. β_3_ and β_4_ for covariate regressors dry weight (w) and protein concentration (p), respectively. x_1_(i) = level of first predictor variable of the i^th^ trial, x_2_(i) = level of second predictor variable in the i^th^ trial, and x_3_(i) and x_4_(i) = levels for w and p, respectively. ε = independent and normally distributed error terms in the i^th^ trial:

$$y\left(i\right)=\beta_{0}+\beta_{1}x_{1}i+\beta_{2}x_{2}i+\beta_{3}x_{3}i+\beta_{4}x_{4}i+\varepsilon i$$

The least squares means are now adjusted for the effect of covariates x_3_ and x_4_ (w and p), and this model used to construct a design matrix for statistical test. In interaction tests, the nominal groups are crossed to each other (products are taken) and incorporated into the design matrix.

Correlations between MEN activity, *Men* expression, and expression of genes of interest were performed using SigmaPlot version 11.0 software (Systat Software, Inc., San Jose, CA).

### Transcription factor binding site (TFBS) predictions

TFBS were predicted using MatInspector with an optimized core matrix similarity of 0.90 (Cartharius *et al.* 2005). TFBS were considered for analysis if found within highly conserved regions as previously predicted by phylogenetic footprinting (Lum and Merritt 2011).

## Results

### *Trans*-interactions at *Men* are pairing dependent (transvection)

**Figure 2:**
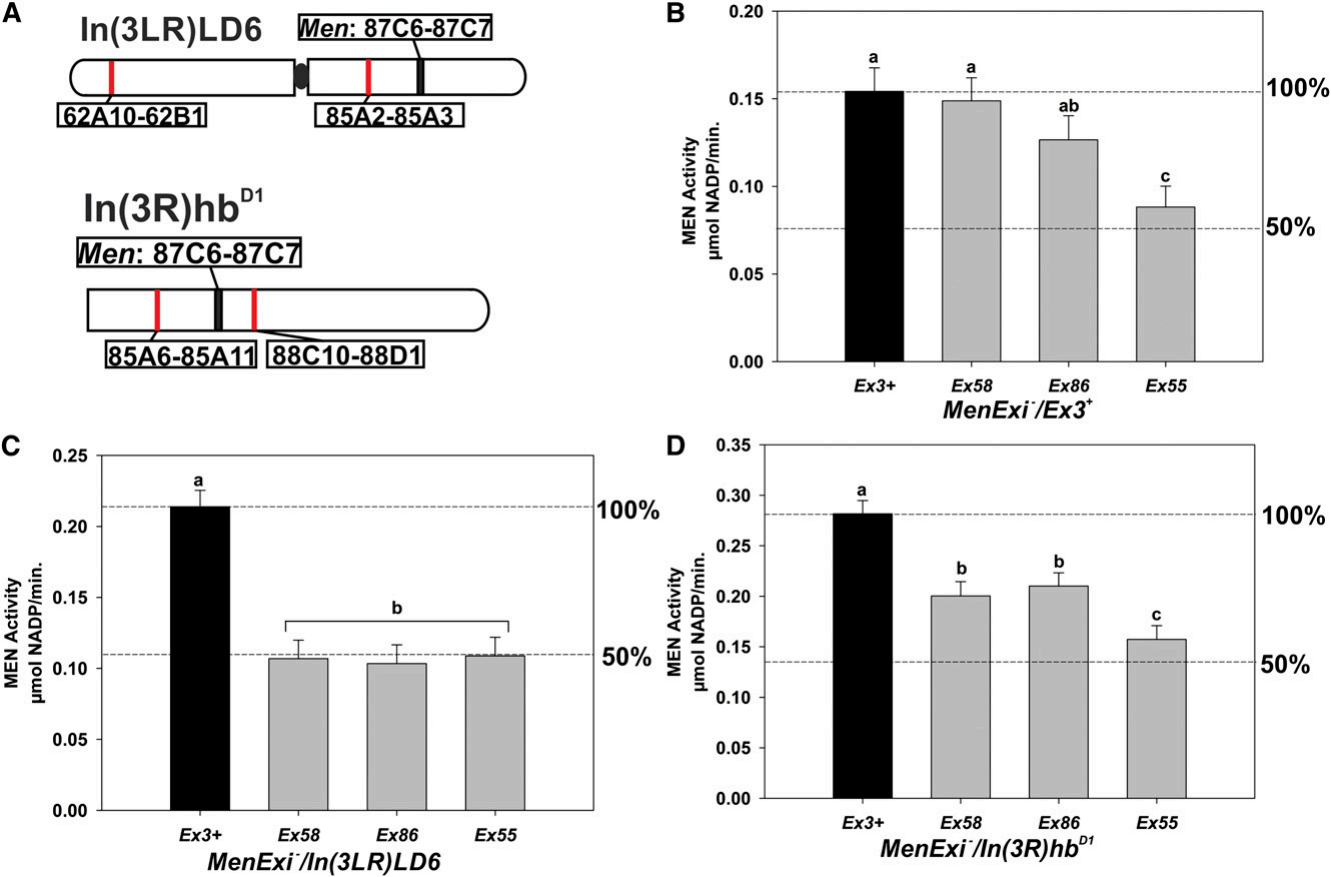
The *trans*-interactions at the Men locus are transvection. MEN activity in flies heterozygous for *MenExi^−^* alleles and either *MenEx3^+^* or inversion chromosomes. (A) Illustration of the third chromosome containing the *In(3LR)LD6* inversion, and the right arm of the third chromosome containing the *In(3R)hb^D1^* inversion. Red lines indicate chromosomal locations of inversion breakpoints. (B) Mean ± SE MEN activity for *MenEx3^+^* homozygotes (100% wild-type activity) and *MenEx3^+^* heterozygotes containing three different *MenExi^−^* alleles. There are significant differences in MEN activity between columns with different letters (*F*_3,15_ = 19.486, *P <* 0.001; Tukey’s honestly significant difference *P* < 0.05). (C) Mean ± SE MEN activity for flies heterozygous for a *MenExi* allele and the *In(3LR)LD6* inversion. The *MenEx3^+^*/*In(3LR)LD6* flies are defined as 100% wild-type activity. There are significant differences in MEN activity between columns with different letters *(F*_3,16_ = 31.210, *P* < 0.001; Tukey’s HSD *P* < 0.05). (D) Mean ± SE MEN activity for flies heterozygous for a *MenExi* allele and the *In (3R)hb^D1^* inversion. The *MenEx3^+^*/ *In (3R)hb^D1^* flies are defined as 100% wild-type activity. There are significant differences in MEN activity between columns with different letters (*F*_3,15_ = 24.313, *P <* 0.001; Tukey’s honestly significant difference *P* < 0.05).

The classic test for transvection uses chromosomal rearrangements to decrease chromosome pairing and disrupt complementation between two mutant alleles, *e.g.*, a promoter-less allele and an enhancer-less allele (reviewed in Duncan 2002). Following these examples, to test whether the *trans*-interactions observed at *Men* were pairing-dependent, *i.e.*, transvection, we created heterozygotes of three of the *MenExi^−^* alleles, and two chromosomes carrying inversions. In the *Men* system, only a single mutant allele is necessary because complementation (up-regulation of *Men*) is tested in *Men^−^*/*Men^+^* heterozygotes, allowing us to cross *Men^−^* alleles with essentially any third chromosome with a functional copy of the *Men* gene (Figure 1, D and E). No complementation was observed in any *MenExi^−^/MenExi^−^* heterozygotes (data not shown). The *In(3LR)LD6* chromosomal inversion is pericentric (extending past the centromere), with the distal breakpoint 62A10-62B1 on 3L, and proximal breakpoint 85A2-85A3 on 3R, between the *Men* locus (87C6-87C7) and the centromere (Figure 2A). The second inversion (*In(3R)hb^D1^*) is paracentric (does not extend past the centromere), with both breakpoints on 3R, 85A6-85A11 and 88C10-88D1, flanking the *Men* locus (Figure 2A). As a control, we created heterozygotes between the *Men^+^* allele (*MenEx3^+^*) and the inversion chromosomes, setting the *MenEx3^+^* heterozygotes as 100% MEN activity for each paired sets of crosses (Figure 2, B−D). *MenEx3^+^* is a perfect excision derived from the same *P*-element line as the *MenExi−* alleles (Merritt *et al.* 2005), meaning that these alleles are exactly the same genetic background.

The *trans*-interactions were eliminated or reduced in the inversion heterozygotes. All *MenExi^−^/MenEx3^+^* heterozygotes have significantly greater that 50% wild-type MEN activity (consistent with previous work; Figure 2B), but, strikingly, all *MenExi^−^/In(3LR)LD6* heterozygotes tested had MEN activity that was essentially 50% that of the *MenEx3^+^/In(3LR)LD6* heterozygotes (Compare Figure 2, B and C; Table S3). The observed loss of up-regulation in heterozygotes with the larger inversion indicates that the *trans*-interactions at the *Men* locus are pairing-dependent, and transvection as classically defined. In contrast, pairing is reduced in some, but not all, of the heterozygotes with the smaller inversion. *MenEx86^−^/In(3R)hb^D1^* and *MenEx55^−^/In(3R)hb^D1^* heterozygotes showed significantly greater than 50% activity, with levels of up-regulation indistinguishable from heterozygotes with the *MenEx^+^* chromosome (compare Figure 2, B and D). Although *MenEx58^−^/In(3R)hb^D1^* heterozygotes had greater than 50% activity, the up-regulation was lower than in the *MenEx58^−^/MenEx3^+^* heterozygotes, a further example of the allele-specific fine scale modulation of *trans*-effects that we have previously observed (Lum and Merritt 2011). In sum, the disruption of up-regulation in MEN activity by a large chromosomal inversion demonstrates that the *trans*-interactions at *Men* are pairing-dependent and therefore represent transvection. The contrast in results between the two inversions suggests that the smaller inversion may modify, but not eliminate, pairing and that the effects of this modification on the *trans*-effects at the locus depend on the mutations involved.

### Transvection at the *Men* locus is sensitive to environmental conditions

Previous evidence suggests that levels of transvection vary with environment, at least cellular environment, *i.e.*, transvection is plastic, sensitive not robust, to changes in environment. To test whether transvection demonstrates environmental plasticity we held sets of adult *MenExi*^-^ heterozygotes at different temperatures and then compared transvection (*i.e.*, up-regulation of MEN activity) with controls. Following Lum and Merritt (2011), we crossed a set of *MenExi^−^* alleles, and the *MenEx3^+^* allele, to a set of five third chromosome genetic backgrounds. Emerging F_1_ heterozygote males were transferred to one of two experimental temperatures, 21° or 29°, or maintained at 25° as a control group, and aged for 4 d. We limited temperature shifts to adult flies (*i.e.*, instead of rearing flies at three temperatures) to reduce rearing effects that could lead to differences in MEN activity or overall metabolism and possibly confounding our results. After the four day exposures, all enzyme assays were done at 25° so that we were measuring changes in available protein, not temperature-driven differences in kinetics.

**Figure 3:**
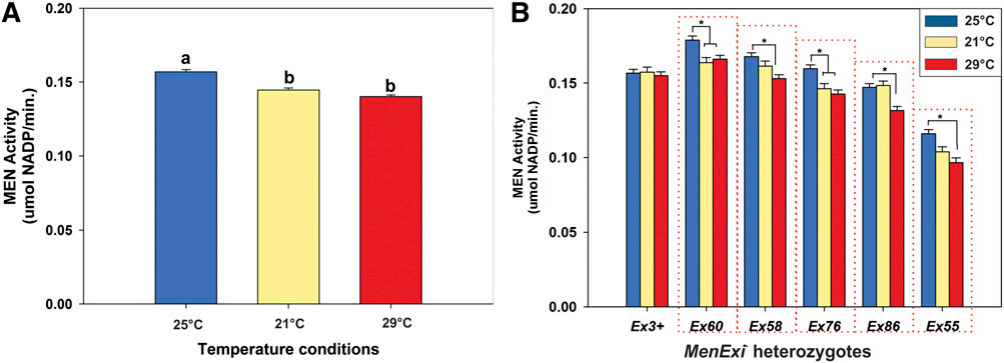
Change in environment (temperature) reduces transvection at Men. (A) Mean ± root mean square error MEN activity of all genotypes, all *MenExi* alleles, across five genetic backgrounds, from each holding temperature: 25° (control; blue bars), 21° (yellow bars), 29° (red bars). Both 21C and 29° groups are significantly lower than the 25° group; see text for exact *P* values. (B) Mean ± SE MEN activity of *MenExi* allele heterozygotes across five genetic backgrounds from flies held at 25°, 21°, and 29°. Asterisks indicate groups that were significantly different according to Tukey’s honestly significant difference test (*F*_10,2288_ *=* 1.775, *P <* 0.001, Tukey’s honestly significant difference *P* < 0.05). Note that only *MenExi^−^* allele heterozygotes (boxed by red dotted line) show significant differences in MEN activity across holding temperatures.

Overall transvection, up-regulation of MEN, was reduced by a change in adult environment. MEN activity was significantly lower in the *MenExi^−^* heterozygotes moved to experimental temperatures (*F*_2,2288_ *=* 46.998, *P <* 0.0001; Figure 3A; Table S3). When examined separately (Figure 3B), MEN activity for each *MenExi−* allele was also generally greater in the 25C flies than either experimental treatment. In contrast, exposure temperatures did not impact MEN activity in the *MenEx3^+^* heterozygotes (Figure 3B, far left columns). MEN activity in the *MenEx3^+^* heterozygotes, with intact promoters at both homologous loci, is likely predominantly regulated by *cis*-interactions, through “*cis*-preference” (Geyer *et al.* 1990; Figure 1D), while MEN activity in the *MenExi^−^* heterozygotes is likely a function of both *cis*- and *trans*-effects (Lum and Merritt 2011). The contrast in results between *MenExi^−^* heterozygotes and *MenExi^−^* heterozygotes suggest that *trans*-, but not *cis*-, based regulation of MEN is sensitive to changes in the environment. We are not suggesting that MEN activity does not respond to changes in temperature, but that the *cis*- and *trans*- components of the regulation of the *Men* gene appear to respond to changes in thermal environment differently.

### Transvection displays genotype by environment (GXE) interactions

**Figure 4:**
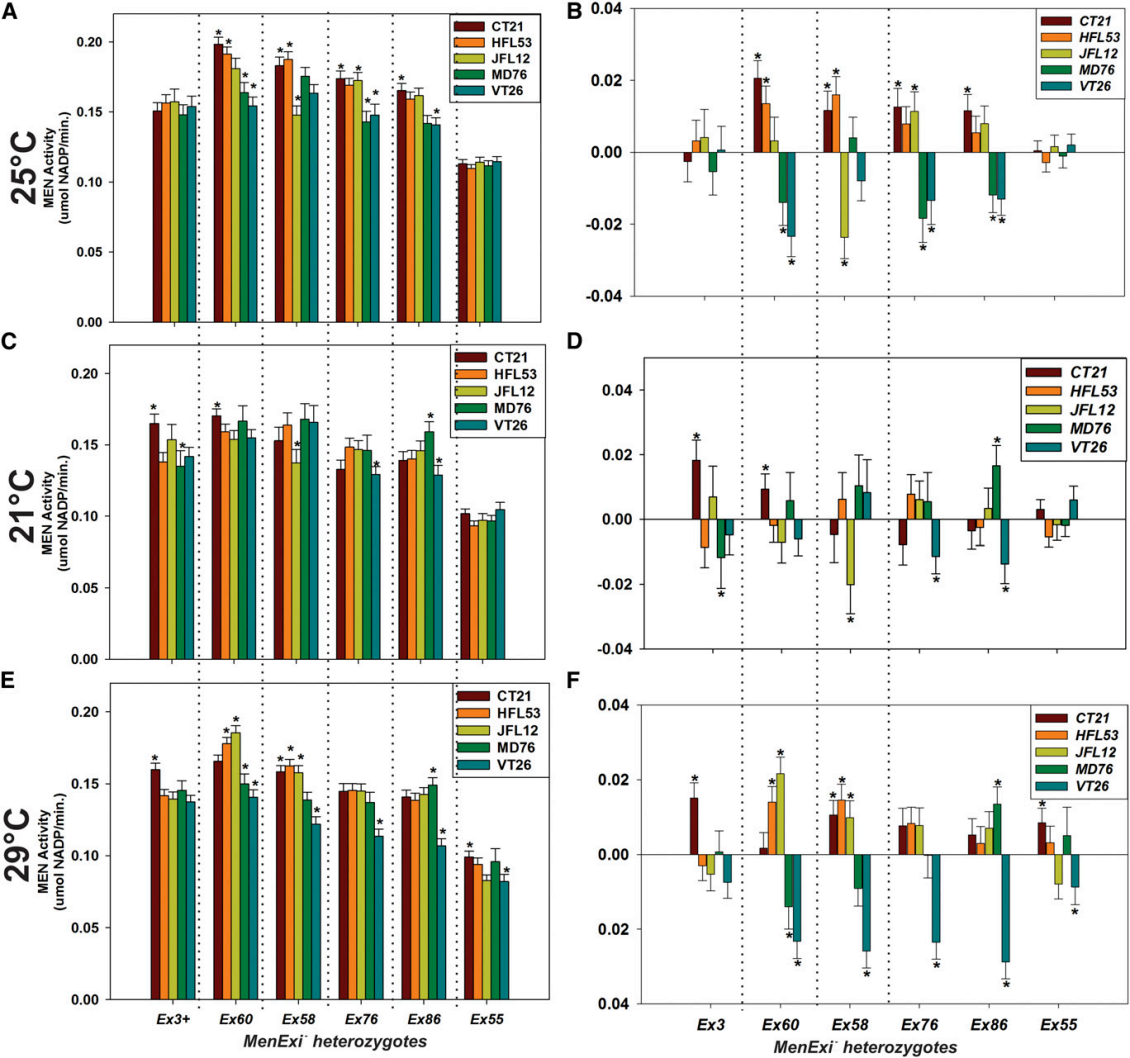
Genetic background significantly modifies transvection at Men. Mean ± root mean square error MEN activity of *MenExi/*isothird chromosome heterozygotes held at (A) 25°, (C) 21°C, or (E) 29°. These same activities, standardized by both average excision allele and third chromosome activities, are shown in (B), (D), and (F), indicating significant interactions between excision alleles and genetic background in flies from all three groups. Asterisks indicate lines that are significantly different from the standardized average at a 0.95 threshold (according to a *t*-test).

The genetic background, excision allele, and background by allele effects on the *trans*-interactions at the *Men* locus are themselves modified by changes in environment. Figure 4 shows MEN activity for each of the *MenExi^−^* heterozygotes across the five genetic backgrounds in flies held at 25°, 21°, or 29°. Consistent with earlier work at 25° (Lum and Merritt 2011), the amount of transvection was sensitive to both the excision allele and genetic background and there were significant interaction effects between excision alleles and genetic backgrounds. To visualize these interactions, we followed Lum and Merritt (2011) and standardized all crosses by both excision allele and background, and looked for statistical outliers (Figure 4, B, D, and F). After standardization, MEN activity in samples that show no interactions will not be significantly different from zero, which represents the average MEN activity of that excision allele by background group. In flies held at 25°, all *MenExi^−^* alleles except *MenEx55^−^* had significant interactions with at least one genetic background (*i.e.*, were significantly different from zero, *F*_20,711_ *=* 2.994, *P* < 0.001; Figure 4B). *MenEx3^+^* heterozygotes, whose regulation should be dominated by *cis*-effects, also showed no deviation across the five backgrounds at 25° (Figure 4B). Comparison of Figure 4, A and B (25°) with Figure 4, C and D (21°) and Figure 4, E and F (29°) shows holding temperature substantially influences these interaction effects. We found significant interactions in both experimental conditions (21°: *F*_20,775_ = 3.456, *P <* 0.001; 29°: *F*_20,800_ = 3.444, *P <* 0.001), but the interactions were visibly different across the three holding temperatures (Figure 4, B, D, and F). Holding temperature also influenced the contribution of the genetic background effects themselves to variation in MEN activity. Genetic background had a significant effect on MEN activity in control flies and flies moved to 29° (*F*_4,800_ *=* 26.967, *P* < 0.001), but not flies moved to 21° (not significant).

Changes in holding temperature also resulted in significant background effects on MEN activity for both *MenEx3^+^* and *MenEx55^−^*; two alleles that we expected, for different reasons, to have minimal *trans*-effects. Although *MenEx55^−^* showed minimal transvection overall (Figure 4), significant interactions were detected in flies held at 29° (Figure 4, E and F). Interestingly, we detected a significant effect of background on the wild-type allele *MenEx3^+^* under the experimental temperatures, but not at 25°. The observed significant background effects on *MenEx3^+^* in flies held at 21° and 29° suggest that changes in environment, here temperature, may expose underlying differences in genetic background not apparent under more constant or benign conditions.

### qRT-PCR reveals correlation between *Men* and *Abd-B* expression

**Figure 5:**
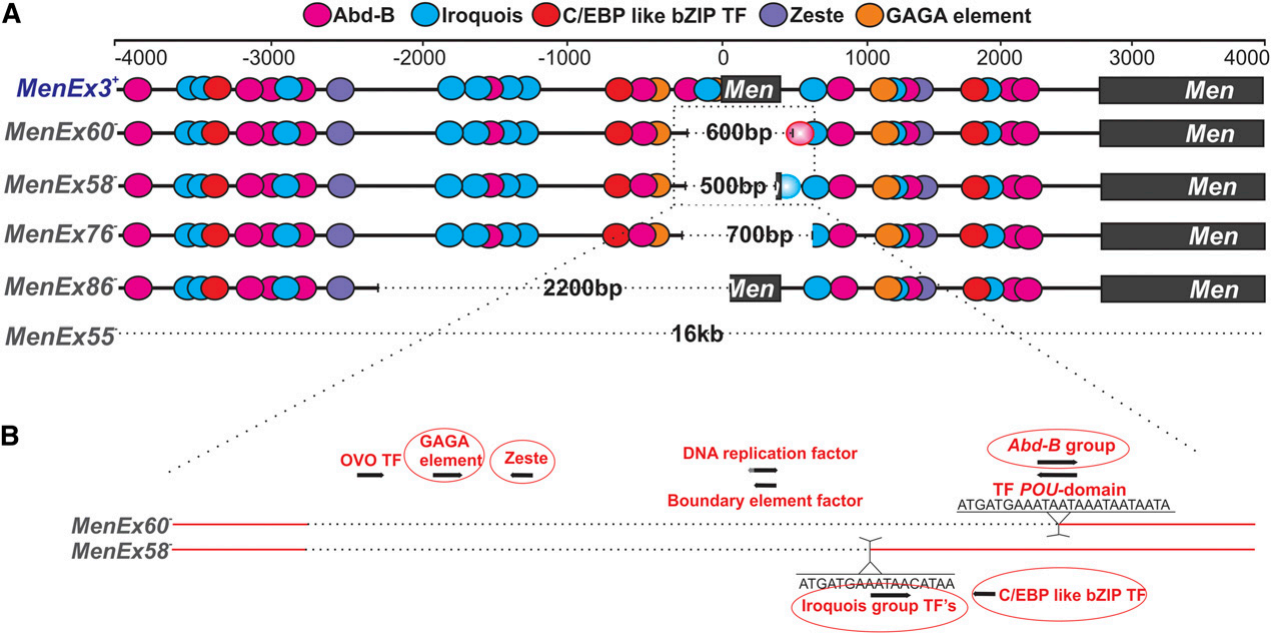
Putative transcription factor binding sites (TFBSs) that may participate in gene regulation and transvection at the Men locus. (A) Colored circles indicate putative TFBSs for the five genes we have analyzed in our study: Abd-B, Iroquois, C/EBP-like bZIP TF, GAGA element, and zeste. For each *MenExi^−^* allele, we indicate the excised region with bracket dotted lines. Faded circles represent TFBSs unique to an excision allele (in *MenEx58^−^* and *MenEx60^−^*). (B) Detail of the excision site of *MenEx58^−^* and *MenEx60^−^*, two alleles that differ in deletion size by ~100 bp and significantly differ in their ability to drive transvection. Each allele has a unique insertion at the excision site: *MenEx58^−^* has an additional Iroquois site; *MenEx60*^−^ has an additional Abd-B site. TFBSs circled in red correspond to transcription factor genes analyzed with quantitative reverse-transcription polymerase chain reaction.

The differences in transvection we observed across excision alleles, genetic backgrounds, and holding temperatures could be functions of the presence or absence of TFBS (*i.e.*, local factors) and/or differences in the activity of TF (*i.e.*, distant factors) between alleles and backgrounds. A number of predicted TFBS near the *Men* transcription start site are deleted, retained, or inserted in different *MenExi^−^* alleles, and the presence or absence of these elements may drive or modify differences in *trans*-activity observed between alleles (Figure 5A; Lum and Merritt 2011). To investigate this possibility, we focused on two excision alleles, *MenEx60^−^* and *MenEx58^−^*: alleles that differ significantly in their ability to drive transvection but only by approximately 100 bp in excision size (Figure 1; Lum and Merritt 2011). *MenEx60^−^* is also of particular interest because it consistently drives greater than 100% wild-type MEN activity when heterozygous with a wild-type chromosome. Figure 5B diagrams the excision sites of these two alleles, indicating TFBS that have high matrix similarity (>0.90 core matrix similarity to optimal transcription factor binding matrix). Two putative TFBSs are found in *MenEx58^−^*, but not *MenEx60^−^*: C/EBP-like bZIP and Iroquois. C/EBP-like bZIP can be bound by the transcription factor Slowbordercells (Slbo), and Iroquois can be bound by Mirror (Bilioni *et al.* 2005; Murphy *et al.* 1995). Additionally, one putative TFBS is found in *MenEx60^−^*, but not *MenEx58^−^*: Abd-B, which can be bound by Abdominal-B protein (Abd-B; Ekker *et al.* 1994). The Iroquois and Abd-B binding sites are not found in the wild-type genomic sequence and result from the *P*-element insertion/excision events. Similar *P*-element remnant sequences have been shown to modify transvection at other loci (*yellow*; Geyer *et al.* 1990; *Gpdh*; Gibson *et al.* 1999). We suspected that the differential transvection ability of these two alleles could be a function of these distinct sites through differential responses of the two alleles to levels of the respective binding proteins. Finally, two predicted TFBS that are found in the wild-type sequence are deleted in both *MenEx60^−^* and *MenEx58*^−^: GAGA element and zeste. GAGA and zeste sites can be bound by Trithorax-like (Trl; van Steensel *et al.* 2003) and zeste (z; Benson and Pirrotta 1988), respectively. Given the differences between *MenEx60^−^* and *MenEx58^−^* in C/EBP like bZIP, Iroquois, and Abd-B, but not GAGA and zeste binding sites, we predicted that, if these binding sites are functional, the differences in transvection observed between the two alleles across the genetic backgrounds and temperatures could correlate with differences in *slbo*, *mirr*, or *Abd-B* expression, but not *Trl* or *z* expression.

**Figure 6:**
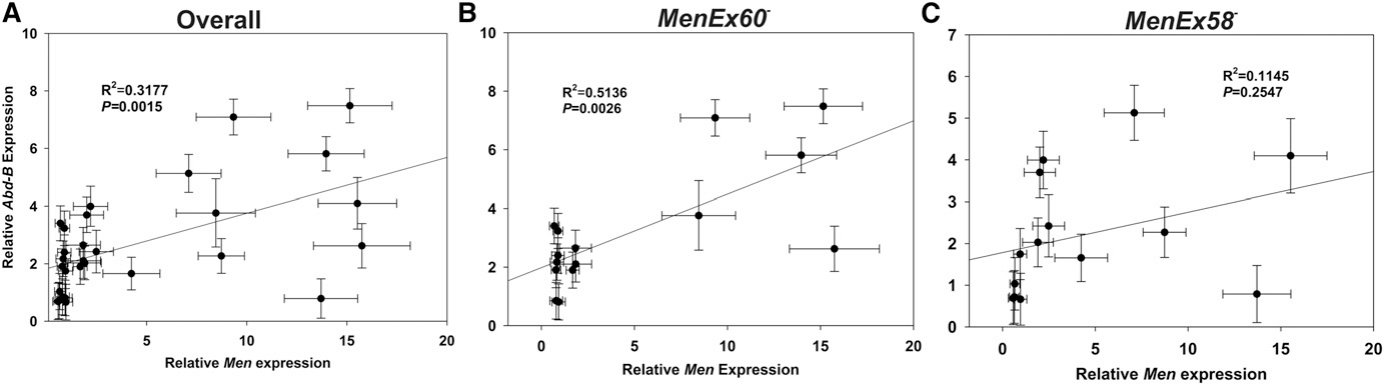
Correlation between Abd-B and Men expression across the whole fly. Mean ± SE. Abd-B *vs.* Men expression in (A) both MenEx60− and MenEx58− heterozygotes, (B) in heterozygotes of MenEx60− alone, and (C) in heterozygotes of MenEx58− alone. Each data point represents expression in a line with a MenExi− allele heterozygous with one genetic background at a single temperature condition (*e.g.*, MenEx58−/CT21 at 25°) and the line is a linear regression of the data. Relative expression of each gene was normalized by the average expression value of that gene across all samples in the experiment (see the *Materials and Methods* section for details of data analysis).

To test for such correlations, we compared relative *Men* expression with the relative expression for five TFs in *MenEx58^−^* and *MenEx60^−^* heterozygotes across the same genetic backgrounds and holding temperature conditions as in the previous set of experiments. Relative expression varied for all genes examined, with the largest differences being between temperature treatments (Figure S1). Of the genes examined, a significant correlation was only apparent between the relative expressions of *Men* and *Abd-B* (R^2^ = 0.318, *P* = 0.015; Figure 6A, Figure S1, Figure S2, and Figure S3). Given the imprecise nature of TF binding and TSBS prediction, it is possible that the predicted sites are nonfunctional, and we cannot rule out the possibility that the sites are bound by other gene products that we did not screen for with qRT-PCR, but nonetheless the lack of correlation in expression between Men and almost all of the genes that we did screen for serves as a reasonable control supporting the significance of the *Men* and *Abd-B* correlation. The trend is apparent in both *MenEx60^−^* and *MenEx58^−^* heterozygotes when examined separately but is only statistically significant in the *MenEx60^−^* heterozygotes (R^2^ = 0.514, *P* < 0.01; Figure 6, B and C), the allele with an additional putative Abd-B binding site (Figure 5B). In both *Men* and *Abd-B*, the largest differences in expression are between holding temperatures, suggesting that in both genes, environment plays a greater role in regulation of expression than genetic background.

**Figure 7:**
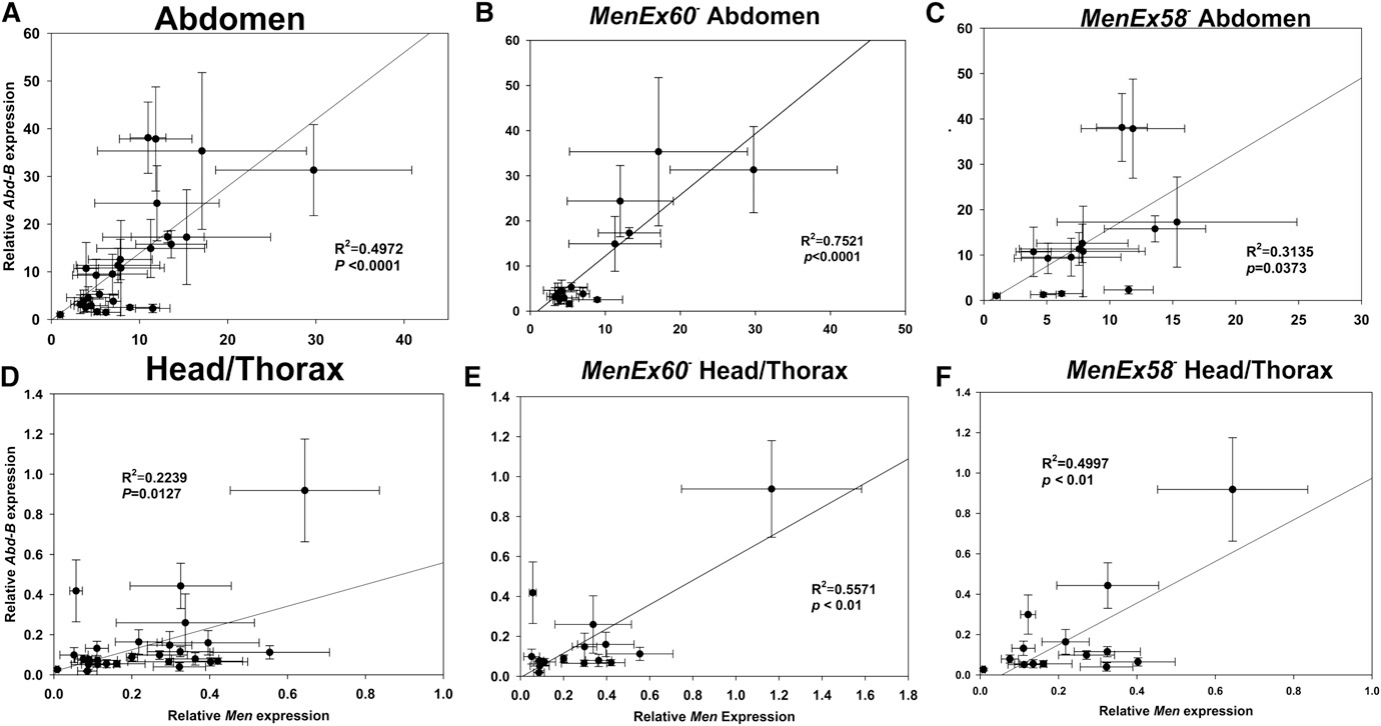
Tissue-specific correlations between Abd-B and Men expression. Mean ± SE. Abd-B and *Men* expression in the abdomen of (A) both *MenEx60^−^* and *MenEx58^−^* heterozygotes, (B) in heterozygotes of *MenEx60^−^* alone, and (C) in heterozygotes of *MenEx58^−^* alone; in the head/thorax of (D) both *MenEx60^−^* and *MenEx58^−^* heterozygotes, (E) in heterozygotes of *MenEx60^−^* alone, and (F) in heterozygotes of *MenEx58^−^* alone. Relative expression of each gene was normalized by the average expression value of that gene across all samples in the experiment.

If differences in *Abd-B* binding are modifying the *trans*-effects at *Men*, then we would expect the correlations between *Men* and *Abd-B* to be strongest in tissues where *Abd-B* expression levels are greatest. In adult flies, *Abd-B* is expressed in greater levels in the abdomen than head/thorax (Chintapalli *et al.* 2007). To test our prediction that correlation will be greater in tissues with greater expression levels, we repeated the previous experiment, measuring only *Men* and *Abd-B* expression and assaying abdomen and head/thorax samples separately. As in the whole fly experiment, expression of both *Abd-B* and *Men* was greatest in flies held at the control temperature, and there was a significant correlation in levels of expression of the two genes (Figure 7). Consistent with our prediction, the strongest correlation was observed in *MenEx60^−^* heterozygotes in abdominal tissues (Figure 7B), which had the greatest levels of expression of both *Adb-B* and *Men*. Expression of the two genes was, however, significantly correlated in all genotypes and in all tissues. The overall correlation in expression, and differences in correlation (although small) between alleles that differ in the number of putative binding sites, are consistent with a model wherein differences in *Abd-B* expression contribute to differences in activation of *Men* expression in *trans* (see the section *Discussion*). Although the correlation between *Men* and *Abd-B* expression could result from coregulation of the two genes by similar mechanisms or pathways, the observation that the pattern is most pronounced in the allele with an additional putative Abd-B site suggests that differences in Abd-B expression may contribute directly to differences in *Men* transvection.

### RNAi knockdown of *Abd-B* reduces MEN activity

**Figure 8:**
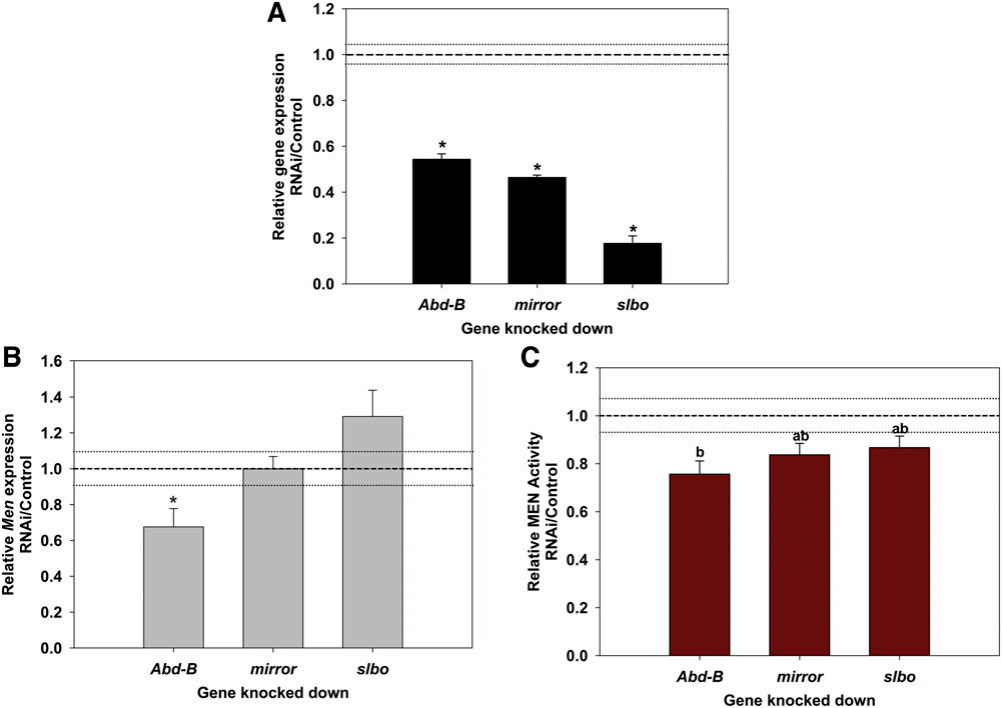
Effect of RNAi knockdown of transcription factors on Men in *cis*. (A) Relative gene expression in all three RNAi experiments showing significant knockdown of expression for all three genes analyzed (asterisk indicates significant reduction in gene expression, *P* < 0.05). (B) *Men* gene expression in *Abd-B*, *mirr*, and *slbo* knocked down lines, relative to control lines. (C) MEN enzyme activity in the same TF knocked down lines, relative to control lines. There are significant differences in MEN activity between columns with different letters (*F*_3,24_ *=* 10.0164, *P <* 0.0001, Tukey’s honestly significant difference *P* < 0.05). In all panels, mean expression/activity of the control lines is indicated by the dotted line with the flanking faint lines indicating standard error.

The predicted TFBSs described previously are present in multiple copies within highly conserved regions across the *Men* locus (Figure 5A; Lum and Merritt 2011) and we suspected that TFs bound to these sites could regulate *Men* expression in *cis*, in addition to any role in *trans*-regulation. Using RNAi, we reduced the expression of three TF genes with binding sites in this region, *Abd-B*, *mirr*, and *slbo*, and assayed for differences in *Men* expression and MEN activity (Figure 8A). *Abd-B* was selected because of its correlation with transvection at *Men* described previously, whereas *mirr* and *slbo* were selected as a contrast because no consistent correlations were found with these genes in *trans*. Following reports of larger RNAi effects with multiple heat shocks (Kristensen *et al.* 2003), vials were heat shocked five times to induce expression of hairpins: 1, 2, 4, 8, and 12 days after egg laying, resulting in reproducible reduction in TF expression (Figure 8). Note that all heat shock treatments were administered postembryogenesis to avoid the primary period during which Abd-B functions in embryonic patterning (Graveley *et al.* 2011, Akam 1987), and that no gross morphological changes were observed in any knockdown flies. Notably, *Men* expression and MEN activity were both significantly reduced only in flies that had *Abd-B* expression knocked down (Figure 8, B and C and Table S3). The reduction in malic enzyme with only a 50% reduction in *Abd-B* may be surprising at first, but previous studies have found malic enzyme to be sensitive to small differences in activity of other genes or the environment (*e.g.*, Merritt *et al.* 2005; Geer *et al.* 1976). These results, and the correlation of *Abd-B* and *Men* expression seen in the previous experiment, support a model in which *Abd-B* plays a role in the regulation of *Men* expression both in *cis* and in *trans*.

## Discussion

Transvection, pairing-dependent modification of gene expression through interactions between sister chromosomes, is itself a complex and plastic phenotype. Here, we have shown that the *trans*-interactions previously documented at the *D. melanogaster Men* locus are transvection and that these effects are sensitive to environment and to background genetic variation. Interestingly, *cis*-interactions at *Men* were more robust to these changes, suggesting that *trans*-interactions are significantly more sensitive to environmental or genetic variation than *cis*-interactions at the same locus. The complexity of interactions, including effects from local and distant genetic variation and the environment, underscores the importance of examining genetic regulation across conditions and genotypes, and the potential for using experimental variability to uncover elaborate mechanisms of molecular regulation.

### *Trans*-interactions at *Men* are pairing-dependent

The *trans*-interaction driven up-regulation of the *Men* locus is significantly reduced or eliminated by changes in chromosomal architecture that to disrupt somatic chromosomal pairing, indicating that these interactions are transvection as classically defined. The elimination of the *trans*-interactions in *MenExi^−^* flies heterozygous for a large pericentric inversion, with breakpoints proximal to the *Men* locus (Figure 2, B and C), is consistent with previous studies using chromosomal rearrangements that disrupt transvection (Gelbart 1982; Golic and Golic 1996; Leiserson *et al.* 1994; Lewis 1954). Interestingly, although the larger inversion eliminates the *trans*-effects, a smaller chromosomal inversion modifies them in a more complicated fashion (Figure 2, B and D); the effects are reduced in the *MenEx58^−^* heterozygotes but not in heterozygotes for either of the other two knockout alleles (compare Figure 1, A and C). These smaller differences in the amount of transvection suggest subtle modulation in *trans*-interactions, and possibly pairing, in response to small or moderate changes in local chromosomal architecture, underscoring the importance and utility of the sensitivity of this MEN system in fine-scale examination of regulation of *trans*-effects and transvection.

The reduction in *trans*-interactions in heterozygotes with either inversion suggests that *Men* has a critical region for pairing extending from at least 85A2-85A3 to 87C6-7 (~4000 kb). Although homologous pairing appears to be initiated at discrete loci (Fung *et al.* 1998), pericentric inversions with one breakpoint between the centromere and the gene of interest can disrupt pairing of structural heterozygotes in *D. melanogaster* (Duncan 2002). Interestingly, genes can differ tremendously in the amount of contiguous flanking homology required to support transvection (the critical region; reviewed by Duncan 2002), and the mechanisms underlying these differences are unclear. Defining the precise extent of the critical region of the *Men* locus awaits examination of a much larger and defined set of rearrangements.

### Transvection is not canalized

Our demonstration that transvection is a plastic phenotype expands the classical view of this mode of gene regulation in *D. melanogaster*, establishing it as a dynamic and variable phenomenon, and opens the door to intriguing questions of variability in *trans*-interactions at other loci and in other systems. Our results suggest that transvection, and by extension somatic chromosomal pairing and other *trans*-interactions, vary not only within tissues (Bateman *et al.* 2012) and across cell types (Mellert and Truman 2012), but also across genetic background and environments. These variations in pairing, and pairing-related *trans*-interactions, also exist across species; differences in chromosomal conformation between *Drosophila* and other organisms have been suggested to reflect a shift in the balance of genes involved in somatic chromosomal pairing (Joyce *et al.* 2012). Further unraveling of the mechanisms underlying this variability will improve our general understanding of the molecular mechanisms of transvection, and *trans*-interactions, in eukaryotes. Changes in environmental condition, an increase or decrease in holding temperature, reduced the overall *trans*-interaction based increases in *Men* expression. We speculate that both the high and low temperature exposures result in some form of stress response leading to changes in chromosomal architecture that reduce the *trans*-interactions, although these response pathways are not necessarily the same. Changes in temperature likely drive complex effects in these organisms, but many of these will be similar in the *Men^+^* and *Men^−^* heterozygotes, our primary focus is on the relative differences in response between these two classes. Exposure to 29° led to upregulation of *Hsp70Aa* expression (Figure S4), suggesting a heat-shock response was elicited by this temperature, consistent with previous results in yeast and *Drosophila* (Gibert *et al.* 2007; Herruer *et al.* 1988; Lindquist 1986; Yao *et al.* 2006). Heat-shock response triggers a genome-wide gene expression response coinciding with chromatin remodelling in a variety of organisms (Gasch *et al.* 2000; Mittal *et al.* 2009; Petesch and Lis 2008; Zhao *et al.* 2005). This remodeling leads to reshuffling of chromosomal architecture, changing access of transcriptional machinery, and modifying gene expression (Aalfs and Kingston 2000). Because transvection is dependent on chromosomal architecture, we suspect that global reshuffling of chromosomes across temperatures leads to a reduction in somatic pairing and thereby the reduction in transvection we observed (Figure 3). The similar reductions in *trans*-interactions in flies exposed to 21° as in the 29° exposed flies suggests that an analogous mechanism may be altering chromosomal architecture at this lower temperature.

Changes in temperature not only reduced transvection but also altered the *MenExi^−^* allele by genetic background interaction effects on transvection. Our results support previous findings that genetic background can significantly modify the amount of *trans*-interactions (Lum and Merritt 2011) and extend these conclusions to show significant genetic background by environment interactions. The significant background by *MenExi^−^* allele (*F*_20, 2288_ = 13.906, *P <* 0.001), and background by allele by temperature interactions (GXE interactions on transvection, *F*_40, 2288_ = 2.5448, *P <* 0.001), suggest that the overall level of transvection is a function of a complex interplay between local and nonlocal genetic effects and the environment. The differences in interaction results at 21° and 29° further suggest that the two temperature exposures are not identical stressors. For example, comparison of the absolute magnitude of background by excision interactions, and the number of significant interactions, shows that genetic background had a much stronger effect on transvection at 29° than at 21° (Figure 4, D and F). These significant interactions underscore the importance of studying mutations across genotypes and environments, and highlight the fact that results from a single background or environment may not necessarily hold true across other backgrounds and conditions.

In contrast, changes in temperature and variation in genetic background had comparatively little influence on *cis*-regulation of *Men*, but changes in temperature did uncover significant interaction effects. *MenEx3^+^* heterozygotes to various genetic backgrounds in this study have two functional copies of the *Men* gene, and based on work from other systems, regulation of expression of either allele is expected to be predominantly in *cis* (Bateman *et al.* 2012; Geyer *et al.* 1990; Morris *et al.* 2004). The *MenEx55^−^* allele shows little transvection, likely because such a large region of the *Men* locus has been removed and, like the *MenEx3^+^* alleles, *cis*-regulation likely predominates over *trans*-effects. Temperature did not affect MEN activity in *MenEx3^+^* heterozygotes, although it did reduce transvection in *MenEx55^−^* heterozygotes. Genetic background had relatively little effect on MEN activity of heterozygotes of either allele at the control temperature (Figure 4B), but significantly modified MEN activity of these heterozygotes at both 21° and 29° (Figure 4, D and F), suggesting that genetic effects controlling *cis*-regulation are also affected by changes in temperature.

Environmental changes can affect TF expression (which we observed, Figure 6, Figure 7, and Figure S1), which in turn can influence global gene expression patterns (Gasch *et al.* 2000; Zhou *et al.* 2012). The background by environment interaction effects we observe, therefore, likely reflect changes in TF expression across environmental conditions that lead to *cis*-regulatory changes observed as changes in MEN activity. This exaggeration of background effects under stressful conditions is consistent with the phenomenon of “cryptic genetic variation,” whereby epistatic interactions are revealed under environmental fluctuations (reviewed by Chandler *et al.* 2013).

### *Abd-B* may regulate *Men* expression in *cis* and in *trans*

*Abd-B* appears to be one such nonlocal element that interacts with both the environment and the local genomic architecture to contribute to the plasticity of transvection observed at the *Men* locus. Changes in the level of transvection in *MenEx60*^-^ heterozygotes were correlated with changes in *Abd-B* expression, which itself was modified by both genetic background and environment. This correlation suggests that Abd-B either has a role in modification of transvection of *Men* or that the two genes are coregulated by similar mechanisms or pathways. The more pronounced effects in flies with an additional Abd-B binding site, that is flies with the *MenEx60^−^* allele, suggests that the former is the most likely explanation. These results suggest that changes in availability of *Abd-B* across background and environment (non-local effect) modify the ability of the *MenEx60^−^* allele to act in *trans* (local effect), leading to differences in the amount of transvection observed across these conditions (GXE interaction on transvection). Given that *MenEx60^−^* consistently shows greater than wild-type levels of MEN activity and is also the only allele that we have recovered with this additional *Abd-B* binding site, it is interesting to speculate that these very high levels of transvection may be, at least in part, driven by the additional site.

In addition to the aforementioned results suggesting *trans*-regulation, our RNAi results suggest that *Abd-B* can regulate, directly or indirectly, *Men* expression in *cis. Abd-B* is a HOX gene in the *Bithorax* (*BX-C*) gene cluster that is regulated via long-range intra-chromosomal interactions mediated by the *Polycomb group* (*PcG*) complexes and chromosomal architecture (Bantignies *et al.* 2011; Tolhuis *et al.* 2011). Previous work has suggested that *Abd-B* expression is sensitive to rearing temperature, and implicated this sensitivity in the phenotypic plasticity of adult abdominal pigmentation in *D. melanogaster* (Gibert *et al.* 2007). Additionally, *Abd-B* interacts with numerous chromatin regulators and may be involved in modulating chromatin architecture (reviewed by Bantignies and Cavalli 2011). We suspect that the correlations we observe between *Abd-B* expression and the amount of transvection at *Men* are the result of similar modulation of chromosomal architecture altering somatic chromosomal pairing in response to temperature-, and possibly genetic background-, driven changes in *Abd-B* expression. *Abd-B* is best known through its role in the regulation of development of the posterior abdominal segments in *D. melanogaster* (Akbari *et al.* 2006). Although the role of *Abd-B* in *Men* regulation is interesting and suggests a novel regulatory function for this TF, it does not explain all the variation seen across all the *MenExi^−^* alleles, genetic backgrounds, and temperatures, and further work is necessary to explore whether changes in additional TFs play a role in the regulation of *Men* expression in *cis* or *trans*.

Transvection is a complex and dynamic phenotype. The previously reported *trans*-interactions at the *D. melanogaster Men* locus are pairing-dependent and are by definition transvection. Further, these interactions are strongly modified by variation in environmental conditions and genetic background, and this plasticity in transvection is associated with changes in TFs coded elsewhere in the genome (*i.e.*, nonlocal factors), in addition to presumed local changes in genomic architecture. We propose that all of these factors interact to modulate transvection through modification of somatic chromosomal pairing. These results strongly suggest that transvection, and *trans*-interactions in general, should be viewed as a dynamic interplay between three factors: local (intragenic), regional or distant (TFs and chromosomal pairing dynamics), and external (environmental conditions). Finally, our findings stress the importance of studying genetic interactions from a dynamic perspective, incorporating both genetic and environmental variation.

## Supplementary Material

Supporting Information
