# Supplementary material for: Transvection-Based Gene Regulation in Drosophila Is a Complex and Plastic Trait
Source: G3 (Bethesda). 2014 Sep 11;4(11):2175–87. doi: 10.1534/g3.114.012484 (PMC4232543; doi:10.1534/g3.114.012484)
Supplement: Supporting Information [file supp_4_11_2175__index.html]

Transvection-Based Gene Regulation in Drosophila Is a Complex and Plastic Trait — Supporting Information 

# Transvection-Based Gene Regulation in *Drosophila* Is a Complex and Plastic Trait

## Supporting Information for Bing *et al.*, 2014

**Files in this Data Supplement:**

- Supporting Information - Figures S1-S4 (PDF, 524 KB)
- Figure S1 - Correlation between *mirr* and *Men* expression. (PDF, 637 KB)
- Figure S2 - Tissue-specific correlations between *mirr* and *Men* expression. (PDF, 598 KB)
- Figure S3 - Correlation between expression of other transcription factors analyzed and *Men*. (PDF, 552 KB)
- Figure S4 - Average relative expression of transcription factor genes across temperatures. (PDF, 236 KB)
