# Supplementary material for: Transvection-Based Gene Regulation in Drosophila Is a Complex and Plastic Trait
Source: G3 (Bethesda). 2014 Sep 11;4(11):2175–87. doi: 10.1534/g3.114.012484 (PMC4232543; doi:10.1534/g3.114.012484)
Supplement: Supporting Information [file supp_g3.114.012484_012484SI.pdf]

G3 · Genes | Genomes | Genetics

Transvection-based gene regulation in *Drosophila* is a complex and plastic trait

Xinyang Bing[1], Teresa Z. Rzezniczak[1], Jack R. Bateman[2], and Thomas J.S. Merritt[1,*]

[1]Department of Chemistry and Biochemistry, Laurentian University, Sudbury, ON, P3E 2C6, Canada

[2]Biology Department, Bowdoin College, Brunswick, Maine 04011

Corresponding Author:
*Thomas J.S. Merritt
Department of Chemistry and Biochemistry
Laurentian University
Sudbury, Ontario P3E 2C6
Phone: 705-675-1151 ext.2189
Fax: 705-675-4844
Email: tmerritt@laurentian.ca

DOI: 10.1534/g3.114.012484

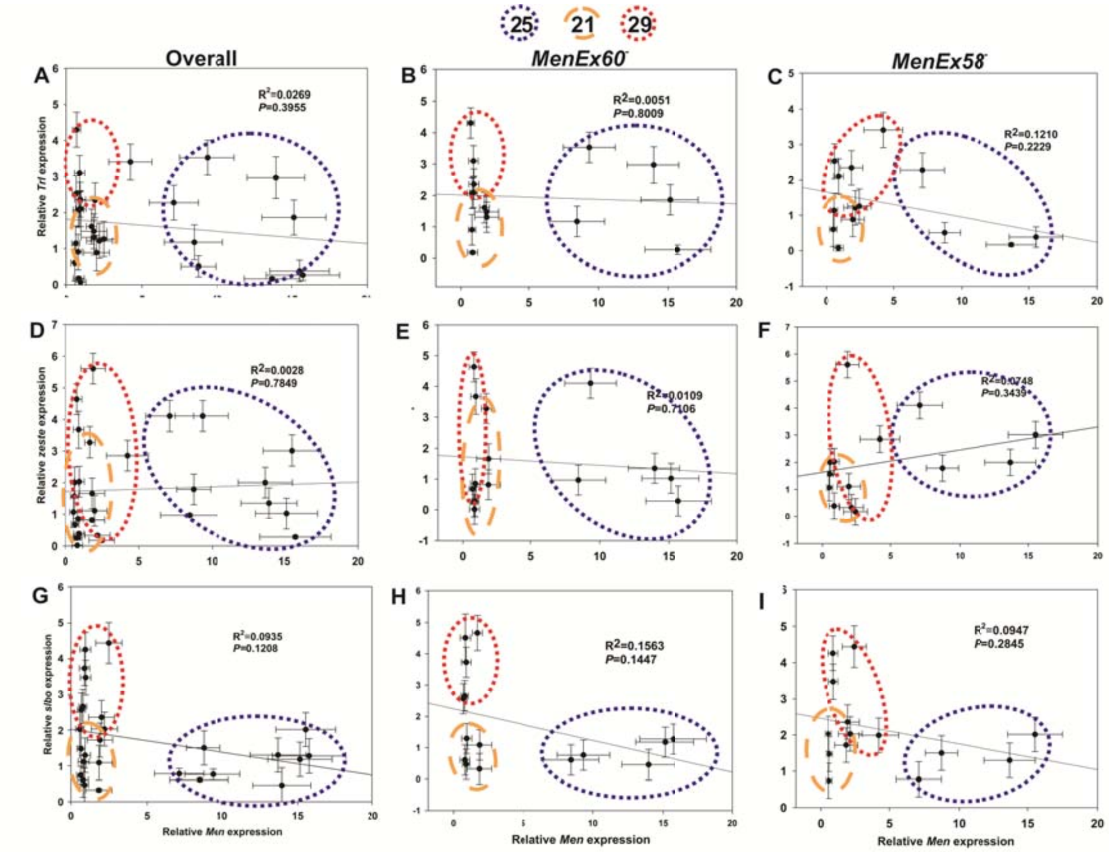

Figure S1 Correlation between *mirr* and *Men* expression*.* *mirr* vs. *Men* gene expression in **(A)** both *MenEx60*⁻ and *MenEx58*⁻ heterozygotes, **(B)** in heterozygotes of *MenEx60*⁻ alone, and **(C)** in heterozygotes of *MenEx58*⁻ alone. Each data point represents expression in a line with a *MenExi*⁻ allele heterozygous with one genetic background at a single temperature condition (*e.g., MenEx58*⁻*/CT21* at 25°C). Relative expression of each gene was normalized by the average expression value of that gene across all samples in the experiment.

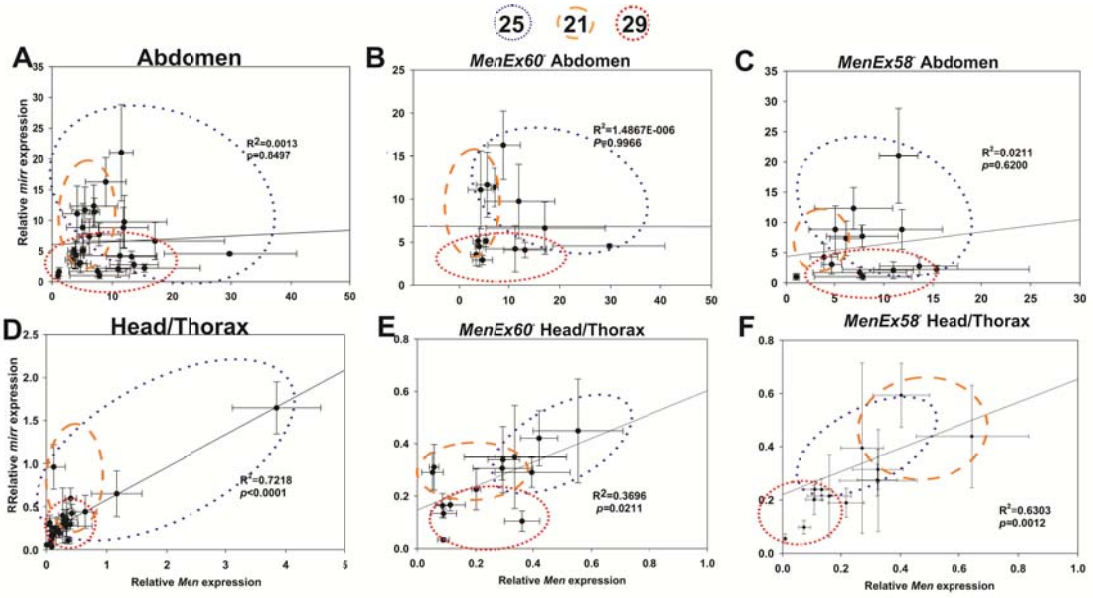

Figure S2 Tissue-specific correlations between *mirr* and *Men* expression. *mirr* and *Men* gene expression in the abdomen of **(A)** both *MenEx60*⁻ and *MenEx58*⁻ heterozygotes, **(B)** in heterozygotes of *MenEx60*⁻ alone, and **(C)** in heterozygotes of *MenEx58*⁻ alone; in the head/thorax of **(D)** both *MenEx60*⁻ and *MenEx58*⁻ heterozygotes, **(E)** in heterozygotes of *MenEx60*⁻ alone, and **(F)** in heterozygotes of *MenEx58*⁻ alone. Relative expression of each gene was normalized by the average expression value of that gene across all samples in the experiment.

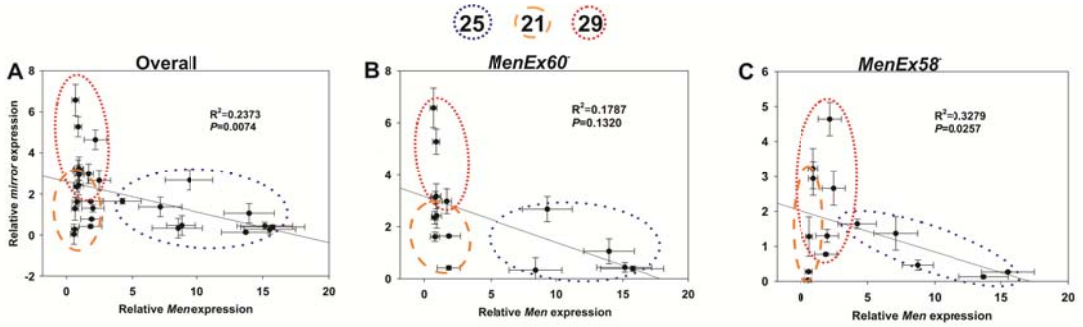

Figure S3 Correlation between expression of other transcription factors analyzed and *Men*. *Trl* vs. *Men* gene expression in **(A)** both *MenEx60*$^-$ and *MenEx58*$^-$ heterozygotes, **(B)** in heterozygotes of *MenEx60*$^-$ alone, and **(C)** in heterozygotes of *MenEx58*$^-$ alone. *zeste* and *Men* expression in **(D)** both *MenEx60*$^-$ and *MenEx58*$^-$ heterozygotes, **(E)** in heterozygotes of *MenEx60*$^-$ alone, and **(F)** in heterozygotes of *MenEx58*$^-$ alone. *slbo* and *Men* expression in **(G)** both *MenEx60*$^-$ and *MenEx58*$^-$ heterozygotes, **(H)** in heterozygotes of *MenEx60*$^-$ alone, and **(I)** in heterozygotes of *MenEx58*$^-$ alone. Relative expression of each gene was normalized by the average expression value of that gene across all samples in the experiment.

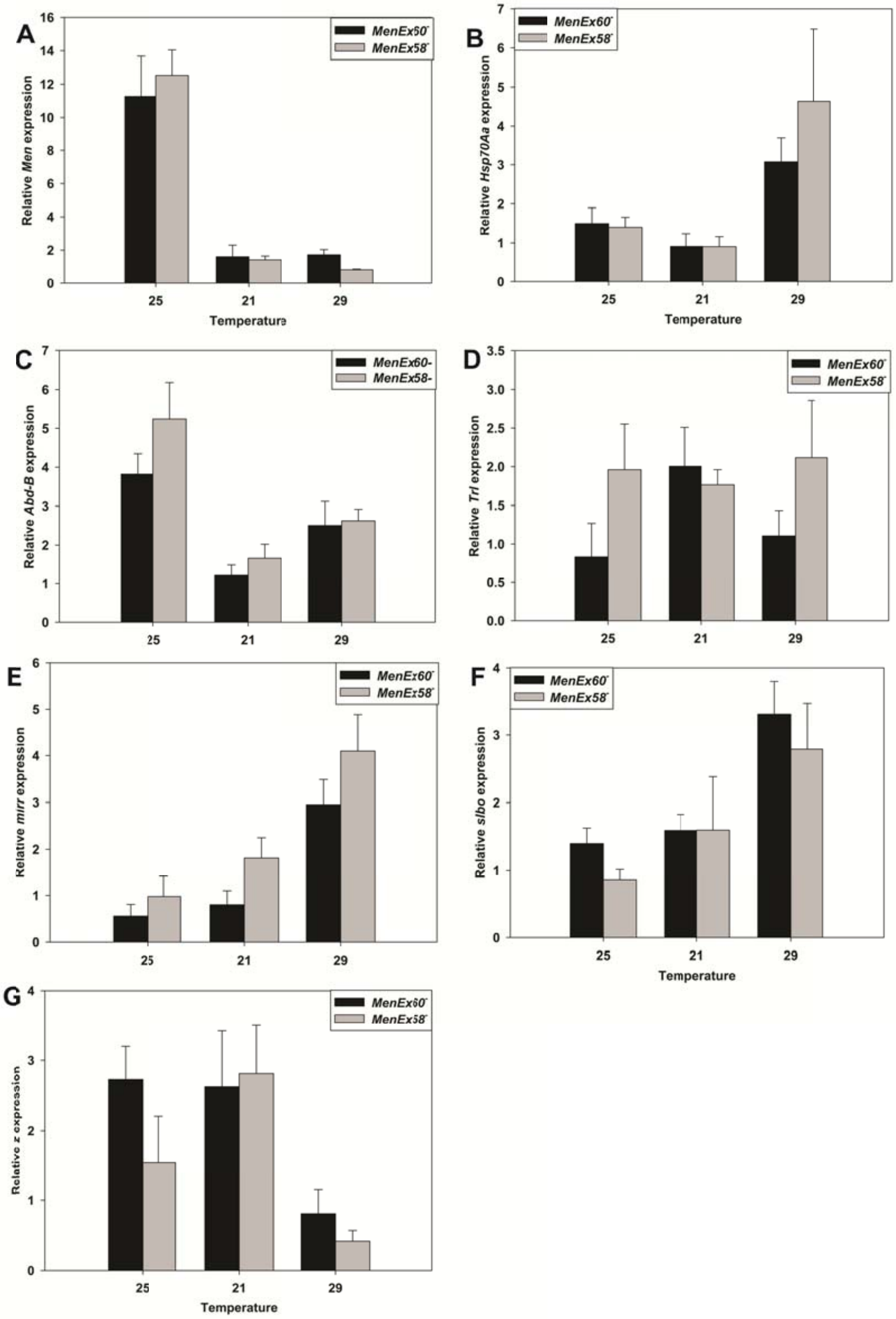

Figure S4 Average relative expression of transcription factor genes across temperatures. Relative gene expression of heterozygotes of each excision allele over five genetic backgrounds: **(A)** *Men,* **(B)** *Hsp70Aa*, **(C)** *Abd-B,* **(D)** *Trl,* **(E)** *mirr,* **(F)** *slbo,* and **(G)** *z.* Relative expression of each gene was normalized by the average expression value of that gene across all samples in the experiment. Temperature significantly affected the expression of all TFs analyzed except for *Trl*: *Hsp70Aa* ($F_{2,89}$ = 16.612, P < 0.001), *Abd-B* ($F_{2,89}$ = 22.350, P < 0.001), *mirr* ($F_{2,89}$ = 46.322, P < 0.001), *slbo* ($F_{2,89}$ = 10.318, P < 0.01), and *z* ($F_{2,89}$ = 6.714, P < 0.05). *Men* expression significantly higher at 25°C than at 21°C or 29°C ($F_{2,89}$ = 89.280, P < 0.001; Figure 7A), *Abd-B* expression, like *Men,* was highest at 25°C (Figure 7C), while *Hsp70Aa, mirr,* and *slbo* expression was highest at 29°C (Figure 7B,E,F). *Abd-B* expression varied significantly across the genetic backgrounds ($F_{4,89}$=21.624, P=0.011).
