# Supplementary material for: Transvection-Based Gene Regulation in Drosophila Is a Complex and Plastic Trait
Source: G3 (Bethesda). 2014 Sep 11;4(11):2175–87. doi: 10.1534/g3.114.012484 (PMC4232543; doi:10.1534/g3.114.012484)
Supplement: Supporting Information [file supp_g3.114.012484_FigureS2.pdf]

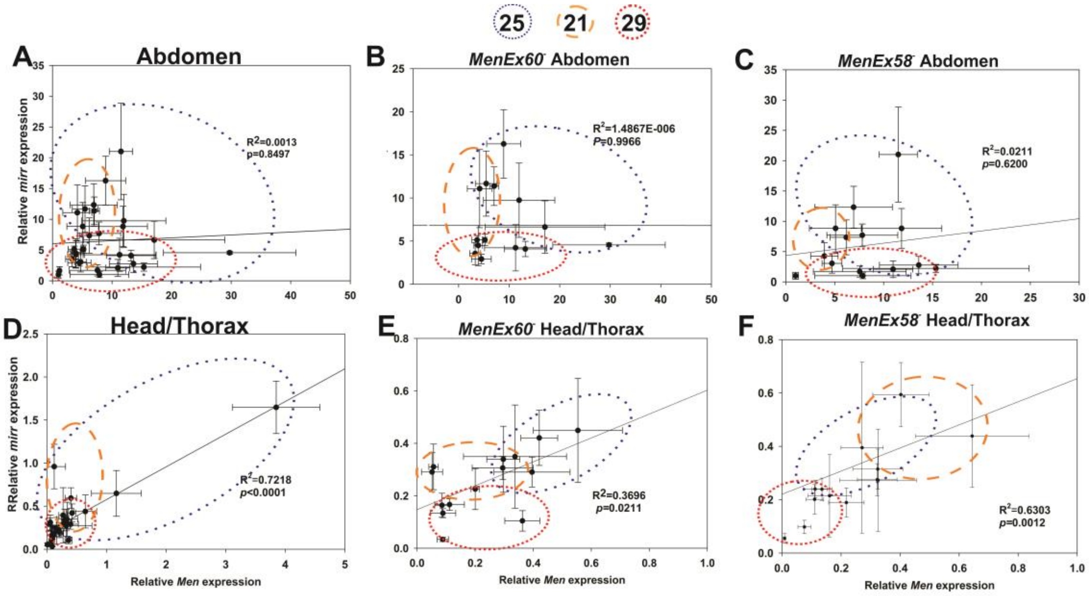

Figure S2 Tissue-specific correlations between *mirr* and *Men* expression. *mirr* and *Men* gene expression in the abdomen of **(A)** both *MenEx60*$^-$ and *MenEx58*$^-$ heterozygotes, **(B)** in heterozygotes of *MenEx60*$^-$ alone, and **(C)** in heterozygotes of *MenEx58*$^-$ alone; in the head/thorax of **(D)** both *MenEx60*$^-$ and *MenEx58*$^-$ heterozygotes, **(E)** in heterozygotes of *MenEx60*$^-$ alone, and **(F)** in heterozygotes of *MenEx58*$^-$ alone. Relative expression of each gene was normalized by the average expression value of that gene across all samples in the experiment.
