# Supplementary material for: Transvection-Based Gene Regulation in Drosophila Is a Complex and Plastic Trait
Source: G3 (Bethesda). 2014 Sep 11;4(11):2175–87. doi: 10.1534/g3.114.012484 (PMC4232543; doi:10.1534/g3.114.012484)
Supplement: Supporting Information [file supp_g3.114.012484_FigureS3.pdf]

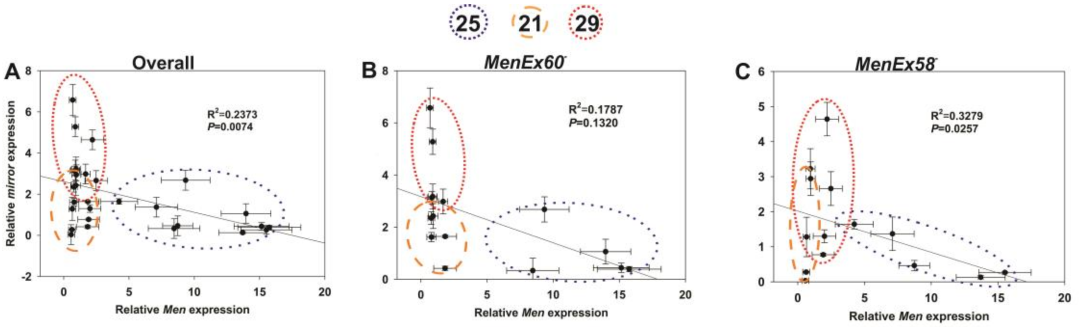

Figure S3 Correlation between expression of other transcription factors analyzed and *Men*. *Trl* vs. *Men* gene expression in **(A)** both *MenEx60*⁻ and *MenEx58*⁻ heterozygotes, **(B)** in heterozygotes of *MenEx60*⁻ alone, and **(C)** in heterozygotes of *MenEx58*⁻ alone. *zeste* and *Men* expression in **(D)** both *MenEx60*⁻ and *MenEx58*⁻ heterozygotes, **(E)** in heterozygotes of *MenEx60*⁻ alone, and **(F)** in heterozygotes of *MenEx58*⁻ alone. *slbo* and *Men* expression in **(G)** both *MenEx60*⁻ and *MenEx58*⁻ heterozygotes, **(H)** in heterozygotes of *MenEx60*⁻ alone, and **(I)** in heterozygotes of *MenEx58*⁻ alone. Relative expression of each gene was normalized by the average expression value of that gene across all samples in the experiment.
