# Supplementary material for: Transvection-Based Gene Regulation in Drosophila Is a Complex and Plastic Trait
Source: G3 (Bethesda). 2014 Sep 11;4(11):2175–87. doi: 10.1534/g3.114.012484 (PMC4232543; doi:10.1534/g3.114.012484)
Supplement: Supporting Information [file supp_g3.114.012484_FigureS4.pdf]

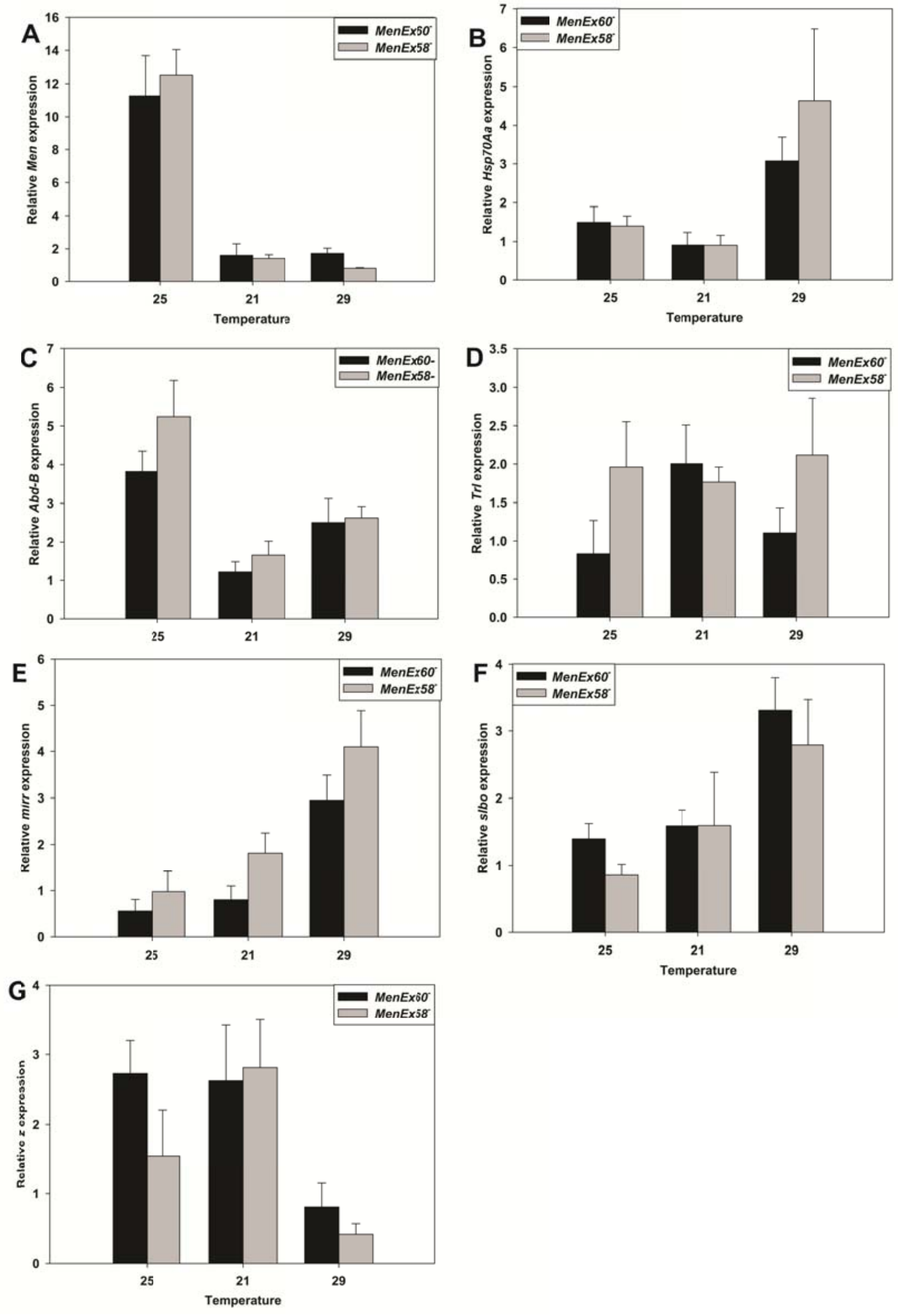


**Figure S4 Average relative expression of transcription factor genes across temperatures.** Relative gene expression of heterozygotes of each excision allele over five genetic backgrounds: **(A)** *Men,* **(B)** *Hsp70Aa*, **(C)** *Abd-B,* **(D)** *Trl,* **(E)** *mirr,* **(F)** *slbo,* and **(G)** *z.* Relative expression of each gene was normalized by the average expression value of that gene across all samples in the experiment. Temperature significantly affected the expression of all TFs analyzed except for *Trl*: *Hsp70Aa* ($F_{2,89}$ = 16.612, $P$ < 0.001), *Abd-B* ($F_{2,89}$ = 22.350, $P$ < 0.001), *mirr* ($F_{2,89}$ = 46.322, $P$ < 0.001), *slbo* ($F_{2,89}$ = 10.318, $P$ < 0.01), and *z* ($F_{2,89}$ = 6.714, $P$ < 0.05). *Men* expression significantly higher at 25°C than at 21°C or 29°C ($F_{2,89}$ = 89.280, $P$ < 0.001; Figure 7A), *Abd-B* expression, like *Men,* was highest at 25°C (Figure 7C), while *Hsp70Aa, mirr,* and *slbo* expression was highest at 29°C (Figure 7B,E,F). *Abd-B* expression varied significantly across the genetic backgrounds ($F_{4,89}$=21.624, $P$=0.011).
